# Assessing psychological flexibility by the Psy-Flex and its relationship with mental health

**DOI:** 10.1192/j.eurpsy.2023.800

**Published:** 2023-07-19

**Authors:** M. Cunha, A. Temido, A. Pinto-Gouveia, A. Galhardo

**Affiliations:** 1 Clinical Psychology, Instituto Superior Miguel Torga; 2 CHUC, Coimbra, Portugal

## Abstract

**Introduction:**

*Psy-Flex* is a brief instrument that assesses psychological flexibility defined, according to the theoretical model of Acceptance and Commitment Therapy, by the set of competencies capable of leading the individual to change behavior, facilitating behaviors that are more adaptive and valued by the individual. This set of psychological skills involved in psychological flexibility is associated with psychological well-being and mental health.

**Objectives:**

The present research sought to translate and adapt *Psy-Flex* to the Portuguese language and, consequently, to conduct the factor analysis, reliability, and validity studies of this instrument in the Portuguese population (in non-clinical and clinical samples)

**Methods:**

The non-clinical sample consisted of 566 individuals (372 female and 192 male) ranging in age from 18 to 74 (M = 36.64; SD = 15.11). The clinical sample included 30 participants aged between 20 and 69 years (M=43.13 and SD= 13.85). The minimum number of years of education is 4, and the maximum is 19 (M=11.80 and SD=3.32). The non-clinical sample was filled out on an *online* platform the protocol that assessed psychological flexibility (Psy-Flex), Psychological Inflexibility and Flexibility (MPFI-24), anxiety and depression symptoms (PHQ-4), and perceived mental health (MHC-SF). In the clinical sample, the protocol was applied individually and face-to-face.

**Results:**

*Psy-Flex* evidenced a unifactorial structure attested to by EFA and CFA. Invariance tests revealed the *Psy-Flex* model to be invariant in configural, metric, and scalar terms for male and female gender and non-clinical and clinical samples. The *Psy-Flex* revealed adequate reliability as assessed by Cronbach’s alpha and Composite Reliability in non-clinical and clinic samples. In non-clinical sample, the *Psy-Flex* showed a positive, moderate to strong, association with flexibility (measured by the MPFI-24-FP) and mental health. It also showed a negative, moderate to strong, association with MPFI-24-IP assessed inflexibility and with depression and anxiety symptoms. Age and years of schooling showed a weak positive association with *Psy-Flex.* Men and women differed significantly, with men showing higher values of psychological flexibility. *Psy-Plex* showed discriminant validity, differentiating between non-clinical and clinical groups. The non-clinical group showed significantly higher values of psychological flexibility.

**Conclusions:**

The present study was innovative in making available a new instrument in the Portuguese language that revealed excellent psychometric characteristics that could be used in community and clinical samples. It also allows the evaluation of efficacy studies of interventions that aim to promote psychological flexibility.

**Disclosure of Interest:**

None Declared

